# IgA *N*- and *O*-glycosylation profiling reveals no association with the pregnancy-related improvement in rheumatoid arthritis

**DOI:** 10.1186/s13075-017-1367-0

**Published:** 2017-07-05

**Authors:** Albert Bondt, Simone Nicolardi, Bas C. Jansen, T. Martijn Kuijper, Johanna M. W. Hazes, Yuri E. M. van der Burgt, Manfred Wuhrer, Radboud J. E. M. Dolhain

**Affiliations:** 1 Department of Rheumatology, Erasmus University Medical Centre, Rotterdam, The Netherlands; 2 Centre for Proteomics and Metabolomics, Leiden University Medical Centre, PO Box 9600, Leiden, 2300 RC The Netherlands

**Keywords:** Rheumatoid arthritis, Pregnancy, Immunoglobulin A, Glycosylation

## Abstract

**Background:**

The Fc glycosylation of immunoglobulin G (IgG) is well known to associate with rheumatoid arthritis (RA) disease activity. The same may be true for other classes of Igs. In the present study, we sought to determine whether the glycosylation of IgA was different between healthy subjects and patients with RA, as well as whether it was associated with RA disease activity, in particular with the pregnancy-associated improvement thereof or the flare after delivery.

**Methods:**

A recently developed high-throughput method for glycoprofiling of IgA1 was applied to affinity-captured IgA from sera of patients with RA (*n* = 252) and healthy control subjects (*n* = 32) collected before, during and after pregnancy.

**Results:**

IgA1 *O*-glycans bore more sialic acids in patients with RA than in control subjects. In addition, levels of bisecting *N*-acetylglucosamine of the *N*-glycans at asparagine 144 were higher in the patients with RA. The levels of several *N*-glycosylation traits were shown to change with pregnancy, similar to what has been shown before for IgG. However, the changes in IgA glycosylation were not associated with improvement or a flare of disease activity.

**Conclusions:**

The glycosylation of IgA differs between patients with RA and healthy control subjects. However, our data suggest only a minor, if any, association of IgA glycosylation with RA disease activity.

**Electronic supplementary material:**

The online version of this article (doi:10.1186/s13075-017-1367-0) contains supplementary material, which is available to authorized users.

## Background

In rheumatoid arthritis (RA), autoantibodies such as rheumatoid factor (RF) and anti-citrullinated peptide antibodies (ACPA) are thought to be crucial not only in initiating the disease process of RA but also in the more chronic stages [1–4]. Representatives of the immunoglobulins G, A and M (IgG, IgA and IgM, respectively) have been recognised for RF as well as ACPA [2, 3, 5]. Besides autoantibodies, sex hormones are thought to have an important role in the pathogenesis of RA, as can be illustrated by the spontaneous improvement of RA during pregnancy [6, 7].

Antibodies are glycoproteins, meaning that they bear at least one glycan on the protein backbone [8]. Glycans may influence immunological properties of antibodies by affecting, for example, receptor binding, half-life, or binding of complement [9, 10]. There are two main classes of glycans described for antibodies, namely the *N*-linked glycans (linked to a nitrogen atom, or *N*) and the *O*-linked glycans (linked to an oxygen atom, or *O*). The *N*-glycans can be found at the asparagine in a known consensus sequence, Asn-Xxx-Ser/Thr, where Xxx can be any amino acid except proline, whereas *O*-linked glycans can be linked to serine or threonine residues, generally in a proline-rich region.

During pregnancy *N*-glycosylation changes have been described for several individual serum proteins, such as alpha-1-antitrypsin, alpha-1-acid glycoprotein, IgG and IgA, as well as for released total serum *N*-glycans [11–15]. For IgG-Fc, bearing almost exclusively *N*-linked glycans, these changes during pregnancy and after delivery were shown to be associated with RA disease activity and the pregnancy-associated improvement thereof [16, 17]. These associations were not observed for the glycans on the variable domain of IgG [18]. For the glycosylation of the other Igs, little is known concerning the association with RA and the pregnancy-associated improvement of RA disease activity. However, given the evident role of antibodies in RA, this information has a high relevance.

Indications for a potential pathogenic role of IgA in RA are given by studies demonstrating the association of IgA RF autoantibodies with bone erosions in RA [19, 20]. Similarly, patients with RA who are seropositive for both IgG- and IgA-ACPA have a more severe disease course than patients with only IgG-ACPA [21].

**Fig. 1:**
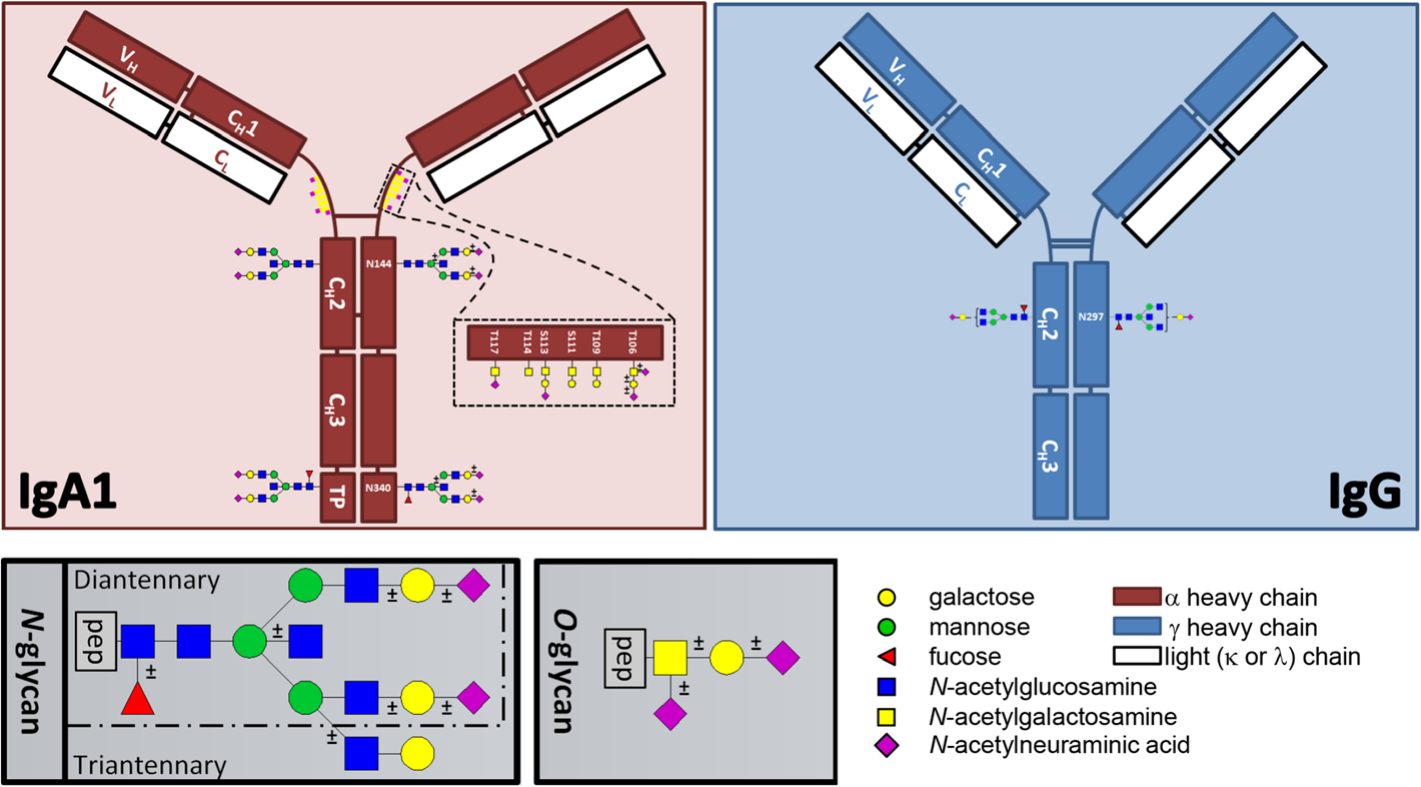
Schematic representation of immunoglobulin A (IgA) and IgG and the sites of their respective glycosylation sites as can be detected by glycopeptide analysis of serum-derived samples. *Insets* show schematic representations of an *O*-glycan, an *N*-glycan and a potential configuration of an *O*-glycopeptide. No linkage information is intended by any position of a monosaccharide

There are two subclasses of IgA, of which IgA1 is the most abundant (approximately 90%) in the human circulation. Whereas most Ig isotypes carry only *N*-linked glycans, the IgA1 subclass carries in addition between three and six *O*-linked glycans [22, 23]. The *O*-glycans are present in the hinge region of IgA1 and consist of one *N*-acetylgalactosamine (GalNAc), which can be decorated by a galactose (Gal) (Fig. 1). Furthermore, sialic acids (SAs) may be attached to either the Gal or the GalNAc. There are two *N*-glycosylation sites on IgA1 (Asn144 and Asn340), and the glycans are of the diantennary complex type, generally consisting of a core of four *N*-acetylglucosamines (GlcNAcs), three mannoses and two Gals. This core structure can be decorated by one or two SAs, one fucose, or one bisecting GlcNAc (Fig. 1) [11, 15, 24–26]. The addition of fucose has been shown to occur only at Asn340 [25]. In addition, low amounts of triantennary glycans can be detected at this site.

Disease-associated IgA *O*-glycosylation changes have been demonstrated in several IgA-related diseases, such as IgA nephropathy, Henoch-Schönlein purpura, Wiskott-Aldrich syndrome and X-linked thrombocytopenia, for which decreased levels of Gal on the *O*-glycans were observed [27–29]. For RA, the published data are less clear, mainly owing to small sample size and to the applied techniques with only a low level of detail. Up to now, it has been suggested that in RA (*n* = 26), the number of Gals on the *O*-glycans is similar to that in control subjects, whereas the level of GalNAc is decreased [30]. The level of GalNAc on the IgA *O*-glycans was found to be associated with the level of galactosylation on the *N*-glycans of IgG. With regard to IgA1 *N*-glycosylation in RA, no differences were found in comparison to healthy individuals (*n* = 5 for both groups) [24].

However, to study the relevance of IgA glycosylation in association with RA disease activity and pregnancy-induced improvement thereof, larger numbers of samples need to be analysed in detail, as we have previously reported for IgG [16]. For this purpose, we recently developed a high-throughput technique for site-specific analysis of both IgA *N*- as well as *O*-glycosylation [15]. By applying this method, we have been able to show pregnancy-associated changes both in *N*- and *O*-glycosylation of IgA in a cohort of healthy women. In the present study, we applied this method to the RA samples collected within the framework of the Pregnancy-induced Amelioration of Rheumatoid Arthritis (PARA) study. The total set consists of approximately 1600 samples obtained from pre-pregnancy onwards from both healthy control subjects and patients with RA. These samples can be used to study differences between healthy control subjects and patients with RA, the association of IgA glycosylation with disease activity in the non-pregnant state, as well as for research on the improvement during pregnancy and flare after delivery and its association with IgA glycosylation.

## Methods

### Study population

The present research is embedded in the PARA study, a nationwide prospective cohort study on pregnancy and RA (*n* = 253) from pre-pregnancy onwards [7, 16]. Healthy control subjects (*n* = 32) were included as a reference group [16]. The study was carried out in compliance with the Helsinki declaration and was approved by the ethics review board at the Erasmus University Medical Centre, Rotterdam, The Netherlands. This study has been described in detail elsewhere [7].

### Categorisation of disease activity and clinical response

Disease activity was assessed using the Disease Activity Score in 28 joints (DAS28) based upon C-reactive protein, as well as swollen and tender joint counts [31]. Responders and non-responders were categorised based upon the European League Against Rheumatism (EULAR) response criteria [32]. The response was defined between the first and third trimesters. A post-partum flare was defined according to the so-called reversed EULAR response criteria [7]. The flare was defined between 6 and 26 weeks post-partum.

### IgA sample preparation and measurement

Site-specific IgA glycosylation analysis was performed as described previously [15]. Briefly, IgA was captured from 10 μl of human serum or plasma using CaptureSelect IgA Affinity Matrix beads (Life Technologies Europe, Bleiswijk, The Netherlands) in 96-well format. After elution from the beads, the samples were dried, followed by overnight digestion at 37 °C with l-1-*p*-tosylamino-2-phenylethyl chloromethyl ketone-treated trypsin (Sigma-Aldrich, Steinheim, Germany) after reduction/alkylation.

Obtained trypsin digestions of IgA samples were enriched for glycopeptides by two-step microtip cotton hydrophilic interaction chromatography-based solid phase extraction, using cotton thread as the solid phase, as described previously [15]. Matrix-assisted laser desorption/ionisation (MALDI) Fourier transform ion cyclotron resonance (FTICR) mass spectrometry (MS) measurements were performed as described elsewhere [15].

### Data extraction and curation

Data Analysis Software 4.0 software package 4 (Bruker Daltonics, Billerica, MA, USA) was used for the visualisation and conversion of the MALDI spectra into.xy files. The XY data of the MALDI-FTICR experiments were internally recalibrated and integrated with a tool developed in-house (MassyTools version 1.6.3.0) [33]. In addition, several quality measures were extracted. After analyte and spectrum curation, several glycosylation traits were calculated as described in Additional file 1: Supplementary Methods.

### Statistical methods

Initial data exploration using SIMCA software (Sartorius Stedim Data Analytics AB, Umeå, Sweden) showed minor batch effects, which were corrected using the ComBat batch correction in R (R Foundation for Statistical Computing, Vienna, Austria). All other statistical tests were performed using Stata/SE 13.1 for Windows software (StataCorp LP, College Station, TX, USA). In the batch-corrected data, we first investigated the differences between patients with RA and healthy control subjects in the non-pregnant state at 6 months post-partum using a Mann-Whitney test. To study which covariates were associated with the levels of the extracted *N*- and *O*-glycosylation traits of patients with RA in the non-pregnant state, multivariable linear regression analysis was performed on the samples obtained at 26 weeks post-partum. Covariates that were studied were the use of prednisone, methotrexate, sulphasalazine, hydroxychloroquine, leflunomide, or tumour necrosis factor (TNF) inhibitors (all different TNF inhibitors grouped together), as well as the DAS28, autoantibody (RF, ACPA or both) seropositivity, and age at delivery.

The effects of pregnancy on the glycosylation traits was studied using multi-level mixed-effects linear regression, similarly to what has been described previously [16], but using Stata software. Comparisons were made between pre-conception and the third trimester (RA only), between the first and third trimesters, between the third trimester and 6 weeks post-partum, between the third trimester and 26 weeks post-partum, and between 6 and 26 weeks post-partum (bisection only). In addition, we investigated possible differences in the pregnancy-associated glycosylation changes between patients who do or do not improve during pregnancy (responders and non-responders), as well as between patients with or without a post-partum flare of disease activity, as described previously [16].

Finally, we explored which glycosylation traits are associated with disease activity at each time point, using a bootstrap approach for a multivariable linear regression model. The pre-conception time point was excluded owing to lack of power. The DAS28 was used as the dependent variable, whereas the use of prednisone, methotrexate, sulphasalazine, hydroxychloroquine, biologics, or leflunomide, autoantibody (RF, ACPA, or both) seropositivity, and age at delivery were treated as fixed elements, and the calculated glycosylation traits (for IgA as obtained in the present study, and for IgG obtained in a previous study [16]) were selected as potential covariates. One thousand rounds of backwards elimination with a *p* < 0.05 cut-off were performed, after which a summary of all models was prepared using the ten most often included glycosylation variables. In all statistical tests, Bonferroni multiple testing correction was performed when applicable, as specified in the presented tables.

## Results

### Study population

Clinical characteristics of patients and healthy control subjects are presented in Table 1.

**Table 1:** Cohort characteristics

	Healthy control subjects (*n* = 32)	Pregnant patients with RA (*n* = 252)						
Mean age at delivery in years (SD)	32.1 (4.4)	32.8 (3.7)						
Median disease duration in years at first visit (range)		4.9 (0.2-28.6)						
ACPA-positive patients, *n* (%)		153/252 (61)						
RF-positive patients, *n* (%)		161/239 (67)						
ACPA- and/or RF-positive patients, *n* (%)		181/252 (72)						
Erosive disease, *n* (%)		150/246 (61)						
Response during pregnancy^a^, *n* (%)		56/120 (47)						
Flare during post-partum period, *n* (%)		69/223 (31)						
Per time point		Pre-conception	First trimester	Second trimester	Third trimester	6 Weeks post-partum	12 Weeks post-partum	26 Weeks post-partum
DAS28, mean (SD)		3.6 (1.1)	3.6 (1.1)	3.6 (1.1)	3.3 (1.1)	3.3 (1.1)	3.6 (1.2)	3.4 (1.1)
Use of prednisone, *n* (%)		37/121 (31)	79/223 (35)	86/234 (37)	81/239 (34)	84/240 (35)	87/242 (36)	78/242 (32)
Use of sulphasalazine, *n* (%)		41/121 (34)	62/223 (28)	64/234 (27)	61/239 (26)	60/240 (25)	72/242 (30)	70/242 (29)
Use of hydroxychloroquine, *n* (%)		9/121 (7)	5/223 (2)	5/234 (2)	4/239 (2)	9/240 (4)	18/242 (7)	17/242 (7)
Use of methotrexate, *n* (%)		0/121 (0)	0/223 (0)	0/234 (0)	0/239 (0)	34/240 (14)	59/242 (24)	74/242 (31)
Use of leflunomide, *n* (%)		0/121 (0)	0/223 (0)	0/234 (0)	0/239 (0)	0/240 (0)	3/242 (1)	4/242 (2)
Use of TNF inhibitors, *n* (%)		5/121 (4)	0/223 (0)	0/234 (0)	0/239 (0)	13/240 (5)	23/242 (10)	29/242 (12)

*Abbreviations: ACPA* Anti-citrullinated peptide antibodies, *DAS28* Disease Activity Score in 28 joints, *RA* Rheumatoid arthritis, *RF* Rheumatoid factor, *TNF* Tumour necrosis factor

^a^The European League Against Rheumatism response criteria require a DAS28 > 3.2 at baseline

### IgA glycosylation in patients with RA and healthy control subjects in the non-pregnant state

#### Site-specific differences in glycosylation between patients and control subjects

The difference between patients with RA and healthy individuals was tested ≥6 months post-partum, when the women had ‘recovered’ from pregnancy, to exclude potentially differential influences of pregnancy on the glycosylation of IgA for patients as compared with control subjects. For the majority of the calculated glycosylation traits, no difference was observed between patients and control subjects (Table 2). However, the number of SAs on the *O*-glycans was significantly higher in patients with RA (3.1, SD 0.18) than in healthy control subjects (3.0, SD 0.14; *p* = 0.0023). For the *N*-glycosylation at Asn144, the level of bisecting GlcNAc was higher in patients (32.0%, SD 8.1%) than in the control subjects (26.3%, SD 4.1%; *p* = 0.0001) (Table 2).

**Table 2:** Glycosylation comparison of healthy control subjects vs. patients with rheumatoid arthritis at the non-pregnant state (≥26 weeks after delivery)

				Healthy		RA		*p* Value
				Mean	SEM	Mean	SEM	
*O*-glycosylation			GalNAc, *n*	4.81	0.009	4.82	0.004	0.230
			Gal, *n*	3.99	0.014	4.03	0.005	0.017
			SAs, *n*	3.05	0.027	3.15	0.013	**0.002**
			SAs per Gal	0.76	0.006	0.78	0.003	0.007
			Gals per GalNAc	0.83	0.003	0.84	0.001	0.028
*N*-glycosylation	**Asn144**		% Sialylation	60.37	0.854	58.09	0.388	0.039
			% Bisection	26.27	0.784	32.04	0.604	**<0.001**
	**Asn340**	Intact	% Sialylation	95.68	0.170	95.17	0.106	0.097
		Trunc.		89.82	0.173	90.13	0.086	0.104
		Intact	% Bisection	57.94	1.057	60.45	0.508	0.046
		Trunc.		56.12	0.893	57.26	0.444	0.355
		Trunc.	% Fucosylation	93.57	0.304	92.98	0.134	0.055
		Trunc.	% Triantennary	5.30	0.298	4.97	0.119	0.136

*Abbreviations*: *Gal* Galactose, *GalNAc N*-acetylgalactosamine, *RA* Rheumatoid arthritis, *SA* Sialic acid, *trunc.* Truncated

Bonferroni-corrected *p* < 0.004 is considered significant, indicated by boldface type

#### Association of IgA glycosylation with clinical variables

To determine which clinical parameters were associated with IgA glycosylation in patients with RA in the non-pregnant state (at 6 months post-partum), multivariable linear regression analyses were performed, using the glycosylation traits as dependent variables and several clinical parameters as independent variables. No models remained significant after Bonferroni correction (*p* < 0.0039). The best model was bisection of the *N*-glycans at Asn340 (trunc.; *p* = 0.0042, *R* ^2^ = 0.12), suggesting positive associations of disease activity (β = 0.21), use of prednisone (β = 0.21) and age at delivery (β = 0.16) (Additional file 2: Table S1) with bisection.

### Pregnancy-associated IgA glycosylation changes

Pregnancy-associated changes were studied using multi-level mixed-effects linear regression models. For the healthy control subjects, the results were similar to those of our previously published work [15], with only minor differences in significance due to a different statistical method and different *p* value cut-off. For the patients with RA, all *N*- and *O*-glycosylation variables, except for sialylation at the intact Asn340, showed pregnancy-associated changes (Additional file 2: Table S2). The *O*-glycans showed an increase in levels of GalNAc during pregnancy, as well as increased levels of Gals, SAs and increased ratios of SAs per Gal and Gals per GalNAc after delivery. All changes were minor (0.3–2% increase). At *N*-glycosylation site Asn144, an increase (1.04-fold) of sialylation was observed during pregnancy, and a decrease (0.96-fold) was observed after delivery. The bisection at this site showed an increase during pregnancy (1.05- up to 1.09-fold), which persisted until 6 weeks post-partum. At Asn340, similar trends were observed, although the effect size for sialylation was tenfold smaller, whereas the pregnancy-associated changes in bisection appear to be slightly more pronounced. Fucosylation, which is not present at Asn144, showed a slight decrease with pregnancy (0.99-fold), and an increase was observed after delivery (1.01-fold). The most pronounced changes were observed for the low levels triantennary glycans at Asn340, which increased 1.10-fold during pregnancy and showed a 0.90-fold down to 0.86-fold decrease post-partum. For all glycosylation traits, the observed changes were highly similar between patients with RA and healthy control subjects.

For the non-truncated Asn340 sialylation, and for Asn144 and truncated Asn340 fucosylation, the mixed model showed no deviating time points after Bonferroni correction (Additional file 2: Table S2). The means for each time point of all traits are depicted in Additional file 2: Table S3 and graphically presented in Additional file 3: Figures S1 and S2.

### IgA glycosylation changes are not different between responders and non-responders

To study whether the changes in IgA glycosylation associate with the improvement of RA disease activity during pregnancy (the response) and the worsening of it post-partum (the flare), Wilcoxon rank-sum tests were performed for each calculated glycosylation trait. No significant differences were identified.

Furthermore, we explored the association of IgA as well as IgG glycosylation traits with disease activity. In the vast majority of the models, IgG glycosylation traits were found to be associated with disease activity. Only at the second and third trimesters of pregnancy was a minor influence of IgA glycosylation observed. More details of the results are described in Additional file 4: Supplementary Results.

## Discussion

This study shows that IgA glycosylation is different between patients with RA and healthy control subjects, and also that pregnancy-associated changes in IgA glycosylation traits occur in the patients in a similar fashion as in the control subjects. However, these changes were not associated with the pregnancy-induced improvement of RA.

Several differences in IgA *N*- and *O*-glycosylation between patients with RA and healthy control subjects were observed. However, these changes showed only minor effect sizes. Previously, it was shown for the IgA *O*-glycosylation that the number of GalNAcs is lower in patients with RA [30]. However, this was not confirmed in our study. The most likely explanation lies in the fact that we were able to observe more high-mass glycopeptides without losing the lower-mass region, causing the differences in the number of GalNAcs (which would be observed mainly in the lower-mass glycopeptides) to lose significance. In contrast, we do show an increased level of SAs on the *O*-glycans in patients with RA (1.03-fold), which would be visible mainly in the high-mass region.

In the present study, we observed hardly any changes with regard to *O*-glycosylation during pregnancy. However, for *N*-glycosylation, we have been able to show that in patients with RA, several pregnancy-associated IgA *N*-glycosylation changes do occur, similar to what we have previously described for healthy control subjects [15]. In addition, IgA *N*-glycosylation appears to show pregnancy-associated changes which are comparable to those observed for IgG-Fc, albeit at different levels [16]. Furthermore, differences between patients with RA and healthy control subjects were observed, with lower levels of *N*-glycan sialylation at Asn144 and increased levels of bisecting GlcNAc on the *N*-glycans at both Asn144 and Asn340. The higher level of bisection has been shown to associate with a less favourable state in several diseases, both on IgG and on cell surface glycans [34, 35]. Interestingly, the pregnancy-associated time lapse for IgA bisection was slightly different from what was observed previously for IgG, with an increase from the first trimester onwards until the first post-partum time point, only after which a decrease starts. For IgG, the levels of bisection were relatively stable during pregnancy and similarly stable (albeit at a higher level) after delivery [16]. Thus, although the general pattern of pregnancy-induced changes in glycosylation of serum IgA and IgG—both produced predominantly by plasma cells—appears to be similar, the functional consequences might be different for IgA compared with IgG and remain to be elucidated.

Finally, we sought to gain more insight into the potential association of IgA glycosylation with RA disease activity. The observed pregnancy-associated changes in glycosylation did not differ between patients whose RA disease activity improved during pregnancy and patients whose disease activity did not improve, nor did they differ between patients with and without a post-partum flare of disease activity. Furthermore, we explored this association using multivariable linear regression analysis, using disease activity as the dependent variable. Models were built with a bootstrap approach of a backwards selection procedure. For four of six time points, we found IgG glycosylation to be the main or even the only significant ‘predictor’. IgA glycosylation was significant only in the second and third trimesters of pregnancy, with quite small effect sizes. Altogether, our data strongly suggest a more prominent association for IgG than for IgA with disease activity, which may imply a more prominent role for changes in IgG glycosylation in the pathogenesis of RA and in the improvement of RA during pregnancy. Nevertheless, the absence of associations of IgA glycosylation with possible effects in the current setting of RA and pregnancy does not exclude any role of IgA glycosylation in IgA functionality.

## Conclusions

We have demonstrated in a large dataset that there is a difference in IgA glycosylation between patients with RA and healthy control subjects, and also that the glycosylation of IgA changes during pregnancy. However, most differences and changes were minor, so it is unlikely that these have any biological significance. In addition, only minor associations of IgA glycosylation with RA disease activity were observed. This all suggests only a limited role for IgA glycosylation in the pathogenesis of RA, which is in sharp contrast to what has previously been shown for IgG glycosylation. Nevertheless, other properties of IgA may still be important in the pathogenesis of RA, such as the level of IgA plasmablasts [36].

## Additional files

Additional file 1: Supplementary methods. Formulae for calculation of IgA glycosylation traits. (DOCX 19 kb)

Additional file 2: Supplementary tables. **Tables S1** Association covariates with glycosylation at non-pregnant state. **Table S2** *p* Values for IgA glycosylation change over time. **Table S3** Mean and SEM values for all calculated traits at all time points. (DOCX 42 kb)

Additional file 3: Supplementary figures. **Figures S1** and **S2** Values depicted in graphs from data in Additional file 2: Table S3. (DOCX 248 kb)

Additional file 4: Supplementary results. Results for statistical comparison of IgG and IgA glycosylation association with disease activity. (DOCX 13 kb)

## Abbreviations

ACPA: Anti-citrullinated peptide antibodies
DAS28: Disease Activity Score in 28 joints
EULAR: European League Against Rheumatism
FTICR: Fourier transform ion cyclotron resonance
Gal: Galactose
GalNAc: *N*-acetylgalactosamine
GlcNAc: *N*-acetylglucosamine
Ig: Immunoglobulin
MALDI: Matrix-assisted laser desorption/ionisation
MS: Mass spectrometry
PARA: Pregnancy-induced Amelioration of Rheumatoid Arthritis study
RA: Rheumatoid arthritis
RF: Rheumatoid factor
SA: Sialic acid
TNF: Tumour necrosis factor
trunc.: Truncated

## References

[1] de Man YA, Bakker-Jonges LE, den Goorbergh CM D-v, Tillemans SP, Hooijkaas H, Hazes JM (2010). Women with rheumatoid arthritis negative for anti-cyclic citrullinated peptide and rheumatoid factor are more likely to improve during pregnancy, whereas in autoantibody-positive women autoantibody levels are not influenced by pregnancy. Ann Rheum Dis.

[2] Rantapää-Dahlqvist S, de Jong BA, Berglin E, Hallmans G, Wadell G, Stenlund H (2003). Antibodies against cyclic citrullinated peptide and IgA rheumatoid factor predict the development of rheumatoid arthritis. Arthritis Rheum.

[3] van der Woude D, Syversen SW, van der Voort EI, Verpoort KN, Goll GL, van der Linden MP (2010). The ACPA isotype profile reflects long-term radiographic progression in rheumatoid arthritis. Ann Rheum Dis.

[4] Gerlag DM, Norris JM, Tak PP (2016). Towards prevention of autoantibody-positive rheumatoid arthritis: from lifestyle modification to preventive treatment. Rheumatology (Oxford).

[5] Lakos G, Soós L, Fekete A, Szabó Z, Zeher M, Horváth IF (2008). Anti-cyclic citrullinated peptide antibody isotypes in rheumatoid arthritis: association with disease duration, rheumatoid factor production and the presence of shared epitope. Clin Exp Rheumatol.

[6] Østensen M, Andreoli L, Brucato A, Cetin I, Chambers C, Clowse ME (2015). State of the art: reproduction and pregnancy in rheumatic diseases. Autoimmun Rev.

[7] de Man YA, Dolhain RJEM, van de Geijn FE, Willemsen SP, Hazes JM (2008). Disease activity of rheumatoid arthritis during pregnancy: results from a nationwide prospective study. Arthritis Rheum.

[8] Arnold JN, Wormald MR, Sim RB, Rudd PM, Dwek RA (2007). The impact of glycosylation on the biological function and structure of human immunoglobulins. Annu Rev Immunol.

[9] Plomp R, Bondt A, de Haan N, Rombouts Y, Wuhrer M (2016). Recent advances in clinical glycoproteomics of immunoglobulins (Igs). Mol Cell Proteomics.

[10] Malhotra R, Wormald MR, Rudd PM, Fischer PB, Dwek RA, Sim RB (1995). Glycosylation changes of IgG associated with rheumatoid arthritis can activate complement via the mannose-binding protein. Nat Med.

[11] Ruhaak LR, Uh HW, Deelder AM, Dolhain RE, Wuhrer M (2014). Total plasma N-glycome changes during pregnancy. J Proteome Res.

[12] van Dijk W, Havenaar EC, der Linden EC B-v (1995). Alpha 1-acid glycoprotein (orosomucoid): pathophysiological changes in glycosylation in relation to its function. Glycoconj J.

[13] Bondt A, Rombouts Y, Selman MHJ, Hensbergen PJ, Reiding KR, Hazes JM (2014). Immunoglobulin G (IgG) Fab glycosylation analysis using a new mass spectrometric high-throughput profiling method reveals pregnancy-associated changes. Mol Cell Proteomics.

[14] Jansen BC, Bondt A, Reiding KR, Lonardi E, de Jong CJ, Falck D (2016). Pregnancy-associated serum *N*-glycome changes studied by high-throughput MALDI-TOF-MS. Sci Rep.

[15] Bondt A, Nicolardi S, Jansen BC, Stavenhagen K, Blank D, Kammeijer GS (2016). Longitudinal monitoring of immunoglobulin A glycosylation during pregnancy by simultaneous MALDI-FTICR-MS analysis of *N*- and *O*-glycopeptides. Sci Rep.

[16] Bondt A, Selman MHJ, Deelder AM, Hazes JM, Willemsen SP, Wuhrer M (2013). Association between galactosylation of immunoglobulin G and improvement of rheumatoid arthritis during pregnancy is independent of sialylation. J Proteome Res.

[17] van de Geijn FE, Wuhrer M, Selman MH, Willemsen SP, de Man YA, Deelder AM (2009). Immunoglobulin G galactosylation and sialylation are associated with pregnancy-induced improvement of rheumatoid arthritis and the postpartum flare: results from a large prospective cohort study. Arthritis Res Ther.

[18] Bondt A, Wuhrer M, Kuijper TM, Hazes JM, Dolhain RJ (2016). Fab glycosylation of immunoglobulin G does not associate with improvement of rheumatoid arthritis during pregnancy. Arthritis Res Ther.

[19] Arnason JA, Jónsson T, Brekkan A, Sigurjónsson K, Valdimarsson H (1987). Relation between bone erosions and rheumatoid factor isotypes. Ann Rheum Dis.

[20] Lindqvist E, Eberhardt K, Bendtzen K, Heinegård D, Saxne T (2005). Prognostic laboratory markers of joint damage in rheumatoid arthritis. Ann Rheum Dis.

[21] Svärd A, Kastbom A, Reckner-Olsson A, Skogh T (2008). Presence and utility of IgA-class antibodies to cyclic citrullinated peptides in early rheumatoid arthritis: the Swedish TIRA project. Arthritis Res Ther.

[22] Takahashi K, Wall SB, Suzuki H, Smith AD, Hall S, Poulsen K (2010). Clustered *O*-glycans of IgA1: defining macro- and microheterogeneity by use of electron capture/transfer dissociation. Mol Cell Proteomics.

[23] Takahashi K, Smith AD, Poulsen K, Kilian M, Julian BA, Mestecky J (2012). Naturally occurring structural isomers in serum IgA1 *O*-glycosylation. J Proteome Res.

[24] Field MC, Amatayakul-Chantler S, Rademacher TW, Rudd PM, Dwek RA (1994). Structural analysis of the *N*-glycans from human immunoglobulin A1: comparison of normal human serum immunoglobulin A1 with that isolated from patients with rheumatoid arthritis. Biochem J.

[25] Tanaka A, Iwase H, Hiki Y, Kokubo T, Ishii-Karakasa I, Toma K (1998). Evidence for a site-specific fucosylation of N-linked oligosaccharide of immunoglobulin A1 from normal human serum. Glycoconj J.

[26] Mattu TS, Pleass RJ, Willis AC, Kilian M, Wormald MR, Lellouch AC (1998). The glycosylation and structure of human serum IgA1, Fab, and Fc regions and the role of *N*-glycosylation on Fcα receptor interactions. J Biol Chem.

[27] Novak J, Julian BA, Mestecky J, Renfrow MB (2012). Glycosylation of IgA1 and pathogenesis of IgA nephropathy. Semin Immunopathol.

[28] Kiryluk K, Moldoveanu Z, Sanders JT, Eison TM, Suzuki H, Julian BA (2011). Aberrant glycosylation of IgA1 is inherited in both pediatric IgA nephropathy and Henoch-Schönlein purpura nephritis. Kidney Int.

[29] Shimizu M, Kanegane H, Wada T, Motoyoshi Y, Morio T, Candotti F (2013). Aberrant glycosylation of IgA in Wiskott-Aldrich syndrome and X-linked thrombocytopenia. J Allergy Clin Immunol.

[30] Wada Y, Tajiri M, Ohshima S (2010). Quantitation of saccharide compositions of *O*-glycans by mass spectrometry of glycopeptides and its application to rheumatoid arthritis. J Proteome Res.

[31] de Man YA, Hazes JMW, van de Geijn FE, Krommenhoek C, Dolhain RJ (2007). Measuring disease activity and functionality during pregnancy in patients with rheumatoid arthritis. Arthritis Rheum.

[32] van Riel PLCM, van Gestel AM, Scott DL, van Riel PLCM, van Gestel AM, Scott DL (2000). Interpreting disease course. EULAR handbook of clinical assessments in rheumatoid arthritis.

[33] Jansen BC, Reiding KR, Bondt A, Hipgrave Ederveen AL, Palmblad M, Falck D (2015). MassyTools: a high throughput targeted data processing tool for relative quantitation and quality control developed for glycomic and glycoproteomic MALDI-MS. J Proteome Res.

[34] Sonneveld ME, Plomp R, Admiraal J, Koeleman C, Hipgrave-Ederveen A, Koelewijn J (2015). IgG alloantibodies against RBC induced by pregnancy or transfusion have unique glycosylation patterns which correlate with clinical outcome of hemolytic disease of the fetus or newborn [abstract]. Blood.

[35] Kohler RS, Anugraham M, López MN, Xiao C, Schoetzau A, Hettich T (2016). Epigenetic activation of *MGAT3* and corresponding bisecting GlcNAc shortens the survival of cancer patients. Oncotarget.

[36] Kinslow JD, Blum LK, Deane KD, Demoruelle MK, Okamoto Y, Parish MC (2016). Elevated IgA plasmablast levels in subjects at risk of developing rheumatoid arthritis. Arthritis Rheumatol.

