# Supplementary material for: IgA N- and O-glycosylation profiling reveals no association with the pregnancy-related improvement in rheumatoid arthritis
Source: Arthritis Res Ther. 2017 Jul 5;19:160. doi: 10.1186/s13075-017-1367-0 (PMC5498977; doi:10.1186/s13075-017-1367-0)
Supplement: Supplementary file 1 — Supplementary methods. Formulae for calculation of IgA glycosylation traits. (DOCX 19 kb) [file 13075_2017_1367_MOESM1_ESM.docx]

Additional File 1

# Supplementary methods

## formulae to calculate the different glycosylation traits.

The following formulae were used to calculate the glycosylation traits for both *N*- and *O*- glycosylation of IgA1, as described before.^1^ For earlier convenience all relative abundances were multiplied by 100. Therefore, the calculated traits resulting in the number of GalNAcs, galactoses or sialic acids were divided by 100. Abbreviations: Q = *O*-glycopeptide; H = hexose; N = *N*-acetylhexosamine; F = fucose; S = sialic acid; R = Asn144 containing *N*-glycopeptide; T = Asn340 containing *N*-glycopeptide; U = truncated Asn340 containing *N*-glycopeptide; _O: oxidized.

Number of *N*-acetylgalactosamines: (3*(Q1H2N3F0S1 + Q1H2N3F0S2 + Q1H3N3F0S1 + Q1H3N3F0S2 + Q1H3N3F0S3)+ 4*(Q1H2N4F0S0 + Q1H2N4F0S1 + Q1H2N4F0S2 + Q1H3N4F0S0 + Q1H3N4F0S1 + Q1H3N4F0S2 + Q1H3N4F0S3 + Q1H3N4F0S4 + Q1H4N4F0S0 + Q1H4N4F0S1 + Q1H4N4F0S2 + Q1H4N4F0S3 + Q1H4N4F0S4 + Q1H4N4F0S5 + Q1H4N4F0S6) + 5*(Q1H2N5F0S1 + Q1H2N5F0S2 + Q1H3N5F0S0 + Q1H3N5F0S1 + Q1H3N5F0S2 + Q1H3N5F0S3 + Q1H3N5F0S4 + Q1H4N5F0S0 + Q1H4N5F0S1 + Q1H4N5F0S2 + Q1H4N5F0S3 + Q1H4N5F0S4 + Q1H4N5F0S5 + Q1H4N5F0S6 + Q1H5N5F0S1 + Q1H5N5F0S2 + Q1H5N5F0S3 + Q1H5N5F0S4 + Q1H5N5F0S5 + Q1H5N5F0S6 + Q1H5N5F0S7) + 6*(Q1H3N6F0S2 + Q1H3N6F0S3 + Q1H4N6F0S1 + Q1H4N6F0S2 + Q1H4N6F0S3 + Q1H4N6F0S4 + Q1H4N6F0S5 + Q1H5N6F0S2 + Q1H5N6F0S3 + Q1H5N6F0S4 + Q1H5N6F0S5 + Q1H6N6F0S4 + Q1H6N6F0S5))/100

Number of galactoses: (2*(Q1H2N3F0S1 + Q1H2N3F0S2 + Q1H2N4F0S0 + Q1H2N4F0S1 + Q1H2N4F0S2 + Q1H2N5F0S1 + Q1H2N5F0S2) + 3*(Q1H3N3F0S1 + Q1H3N3F0S2 + Q1H3N3F0S3 + Q1H3N4F0S0 + Q1H3N4F0S1 + Q1H3N4F0S2 + Q1H3N4F0S3 + Q1H3N4F0S4 + Q1H3N5F0S0 + Q1H3N5F0S1 + Q1H3N5F0S2 + Q1H3N5F0S3 + Q1H3N5F0S4 + Q1H3N6F0S2 + Q1H3N6F0S3) + 4*(Q1H4N4F0S0 + Q1H4N4F0S1 + Q1H4N4F0S2 + Q1H4N4F0S3 + Q1H4N4F0S4 + Q1H4N4F0S5 + Q1H4N4F0S6 + Q1H4N5F0S0 + Q1H4N5F0S1 + Q1H4N5F0S2 + Q1H4N5F0S3 + Q1H4N5F0S4 + Q1H4N5F0S5 + Q1H4N5F0S6 + Q1H4N6F0S1 + Q1H4N6F0S2 + Q1H4N6F0S3 + Q1H4N6F0S4 + Q1H4N6F0S5) + 5*(Q1H5N5F0S1 + Q1H5N5F0S2 + Q1H5N5F0S3 + Q1H5N5F0S4 + Q1H5N5F0S5 + Q1H5N5F0S6 + Q1H5N5F0S7 + Q1H5N6F0S2 + Q1H5N6F0S3 + Q1H5N6F0S4 + Q1H5N6F0S5) + 6*(Q1H6N6F0S4 + Q1H6N6F0S5))/100

Number of sialic acids: (1*(Q1H2N3F0S1 + Q1H2N4F0S1 + Q1H2N5F0S1 + Q1H3N3F0S1 + Q1H3N4F0S1 + Q1H3N5F0S1 + Q1H4N4F0S1 + Q1H4N5F0S1 + Q1H4N6F0S1 + Q1H5N5F0S1) + 2*(Q1H2N3F0S2 + Q1H2N4F0S2 + Q1H2N5F0S2 + Q1H3N3F0S2 + Q1H3N4F0S2 + Q1H3N5F0S2 + Q1H3N6F0S2 + Q1H4N4F0S2 + Q1H4N5F0S2 + Q1H4N6F0S2 + Q1H5N5F0S2 + Q1H5N6F0S2) + 3*(Q1H3N3F0S3 + Q1H3N4F0S3 + Q1H3N5F0S3 + Q1H3N6F0S3 + Q1H4N4F0S3 + Q1H4N5F0S3 + Q1H4N6F0S3 + Q1H5N5F0S3 + Q1H5N6F0S3) + 4*(Q1H3N4F0S4 + Q1H3N5F0S4 + Q1H4N4F0S4 + Q1H4N5F0S4 + Q1H4N6F0S4 + Q1H5N5F0S4 + Q1H5N6F0S4 + Q1H6N6F0S4) + 5*(Q1H4N4F0S5 + Q1H4N5F0S5 + Q1H4N6F0S5 + Q1H5N5F0S5 + Q1H5N6F0S5 + Q1H6N6F0S5) + 6*(Q1H4N4F0S6 + Q1H4N5F0S6 + Q1H5N5F0S6) + 7*(Q1H5N5F0S7))/100

Ratio of sialic acids per galactose: ‘Number of sialic acids’ / ‘Number of galactoses’

Ratio of galactoses per GalNAc: ‘Number of galactoses‘ / ‘Number of GalNAcs’

Asn144 sialylation: 0.5*(R1H5N4F0S1 + R1H5N5F0S1) + 1*(R1H5N4F0S2 + R1H5N5F0S2)

Asn144 bisection: R1H5N5F0S0 + R1H5N5F0S1 + R1H5N5F0S2

Asn340 sialylation: 0.5*(T1H5N4F1S1_O1) + 1*(T1H5N4F1S2_O1 + T1H5N5F1S2_O1)

Asn340 bisection: T1H5N5F1S2_O1

Truncated Asn340 diantennary sialylation: ((0.5*(U1H4N4F0S1 + U1H5N4F0S1 + U1H5N4F0S1_O1 + U1H5N4F1S1 + U1H5N4F1S1_O1 + U1H5N5F0S1 + U1H5N5F0S1_O1 + U1H5N5F1S1 + U1H5N5F1S1_O1) + 1*(U1H5N4F0S2_O1 + U1H5N4F1S2 + U1H5N4F1S2_O1 + U1H5N5F0S2 + U1H5N5F1S2 + U1H5N5F1S2_O1))/(U1H4N4F0S1 + U1H5N4F0S1 + U1H5N4F0S1_O1 + U1H5N4F0S2_O1 + U1H5N4F1S0_O1 + U1H5N4F1S1 + U1H5N4F1S1_O1 + U1H5N4F1S2 + U1H5N4F1S2_O1 + U1H5N5F0S1 + U1H5N5F0S1_O1 + U1H5N5F0S2 + U1H5N5F1S0_O1 + U1H5N5F1S1 + U1H5N5F1S1_O1 + U1H5N5F1S2 + U1H5N5F1S2_O1))*100

Truncated Asn340 diantennary fucosylation: ((U1H5N4F1S0_O1 + U1H5N4F1S1 + U1H5N4F1S1_O1 + U1H5N4F1S2 + U1H5N4F1S2_O1 + U1H5N5F1S0_O1 + U1H5N5F1S1 + U1H5N5F1S1_O1 + U1H5N5F1S2 + U1H5N5F1S2_O1)/(U1H4N4F0S1 + U1H5N4F0S1 + U1H5N4F0S1_O1 + U1H5N4F0S2_O1 + U1H5N4F1S0_O1 + U1H5N4F1S1 + U1H5N4F1S1_O1 + U1H5N4F1S2 + U1H5N4F1S2_O1 + U1H5N5F0S1 + U1H5N5F0S1_O1 + U1H5N5F0S2 + U1H5N5F1S0_O1 + U1H5N5F1S1 + U1H5N5F1S1_O1 + U1H5N5F1S2 + U1H5N5F1S2_O1))*100

Truncated Asn340 diantennary bisection: ((U1H5N5F0S1 + U1H5N5F0S1_O1 + U1H5N5F0S2 + U1H5N5F1S0_O1 + U1H5N5F1S1 + U1H5N5F1S1_O1 + U1H5N5F1S2 + U1H5N5F1S2_O1)/(U1H4N4F0S1 + U1H5N4F0S1 + U1H5N4F0S1_O1 + U1H5N4F0S2_O1 + U1H5N4F1S0_O1 + U1H5N4F1S1 + U1H5N4F1S1_O1 + U1H5N4F1S2 + U1H5N4F1S2_O1 + U1H5N5F0S1 + U1H5N5F0S1_O1 + U1H5N5F0S2 + U1H5N5F1S0_O1 + U1H5N5F1S1 + U1H5N5F1S1_O1 + U1H5N5F1S2 + U1H5N5F1S2_O1))*100

Truncated Asn340 triantennary: U1H6N5F1S1_O1 + U1H6N5F1S3_O1 + U1H6N6F0S0_O1
