# Supplementary material for: IgA N- and O-glycosylation profiling reveals no association with the pregnancy-related improvement in rheumatoid arthritis
Source: Arthritis Res Ther. 2017 Jul 5;19:160. doi: 10.1186/s13075-017-1367-0 (PMC5498977; doi:10.1186/s13075-017-1367-0)
Supplement: Supplementary file 2 — Supplementary tables. Tables S1 Association covariates with glycosylation at non-pregnant state. Table S2 p Values for IgA glycosylation change over time. Table S3 Mean and SEM values for all calculated traits at all time points. (DOCX 42 kb) [file 13075_2017_1367_MOESM2_ESM.docx]

| **Supplementary Table S1 Association of clinical covariates with IgA glycosylation at six months postpartum in RA-patients**. Bonferroni correction for multiple testing was performed for each glycotrait, resulting in an α = 0.05/13 = 0.0039 as significance threshold. When a model is significant the individual variables can be used with α = 0.05. Part **A** gives the summary from the analysis, providing the number of pregnancies included, as well as the significance and R^2^ of the models. Part **B** shows the outcome of the model for bisection of the *N*-glycans at Asn340 (trunc.), which is closest to significance. | | | | | | | | | | |
| --- | --- | --- | --- | --- | --- | --- | --- | --- | --- | --- |
| **A** |  | |  | Model | | |  | **B** |  |  |
|  |  |  |  | n | p | R^2^ |  |  | β | p |
| *O*-glycosylation | |  | # GalNAc^*^ | 200 | 0.099 | 0.07 |  | Prednisone | 0.210 | **0.006** |
|  |  |  | # Gal | 200 | 0.358 | 0.05 |  | Sulfasalazine | -0.006 | 0.939 |
|  |  |  | # SA | 200 | 0.350 | 0.05 |  | Hydroxychloroquine | 0.010 | 0.894 |
|  |  |  | SA per Gal | 200 | 0.317 | 0.05 |  | Methotrexate | -0.081 | 0.271 |
|  |  |  | Gal per GalNAc | 200 | 0.018 | 0.10 |  | Leflunomide | -0.086 | 0.228 |
| *N*-glycosylation | **Asn144** |  | % SA | 168 | 0.192 | 0.07 |  | TNF-inhibitors | -0.075 | 0.310 |
|  |  |  | % Bis | 168 | 0.394 | 0.06 |  | Autoantibody positivity (ACPA, RF, or both) | -0.040 | 0.581 |
|  | **Asn340** | intact | % SA | 193 | 0.455 | 0.05 |  | Age at delivery | 0.163 | **0.024** |
|  |  | trunc. |  | 189 | 0.380 | 0.05 |  | DAS28-CRP | 0.208 | **0.004** |
|  |  | intact | % Bis | 193 | 0.173 | 0.07 |  |  |  |  |
|  |  | trunc. |  | 189 | 0.004 | 0.12 |  |  |  |  |
|  |  | trunc. | % Fuc | 189 | 0.836 | 0.03 |  |  |  |  |
|  |  | trunc. | % Tri | 189 | 0.199 | 0.07 |  |  |  |  |

Additional File 3

^*^ Abbreviations used: GalNAc = *N*-acetylgalactosamine; Gal = galactose; SA = sialic acid; Bis = Bisection; Fuc = fucosylation; Tri = triantennary glycans.

| **Supplementary Table S2** P-values derived from multilevel mixed-effects linear regression analysis of all IgA *N*- and *O*-glycosylation traits for both RA patients (cases) and healthy controls. A p-value of 0.05/13=0.004 was used as significance threshold. Significant findings are indicated in bold font. Because of the unusual time lapse for bisection of the *N*-glycans an additional time window was included for testing. | | | | | | | | | | | | | |
| --- | --- | --- | --- | --- | --- | --- | --- | --- | --- | --- | --- | --- | --- |
|  | *O*-glycosylation | | | | | *N*-glycosylation | | | | | | | |
|  |  |  |  |  |  | Asn144 | | Asn340 | | | | | |
|  | # GalNAc | # Gal | # SA | SA per Gal | Gal per  GalNAc | SA | Bis | SA | | Bis | | Fuc | Tri |
|  |  |  |  |  |  |  |  | intact | trunc | intact | trunc | trunc | trunc |
| pre-conc.^*^ to 3rd trim (cases) | 0.043 | 0.725 | 0.619 | 0.593 | 0.052 | **0.000 ↑** | **0.001 ↑** | 0.366 | 0.025 | 0.756 | 0.021 | **0.003 ↓** | 0.014 |
| 1st trim to 3rd trim (cases) | **0.001 ↑** | 0.181 | 0.171 | 0.033 | 0.473 | 0.092 | **0.000 ↑** | **0.000 ↑** | 0.829 | **0.000 ↑** | **0.000 ↑** | 0.051 | 0.128 |
| 3rd trim to 6 wk pp (cases) | 0.289 | **0.000** **↑** | 0.032 | 0.397 | **0.000 ↑** | **0.000 ↓** | **0.000 ↑** | 0.372 | **0.000 ↓** | **0.000 ↑** | **0.000 ↑** | **0.003 ↑** | 0.013 |
| 3rd trim to 26 wk pp (cases) | 0.410 | **0.001 ↑** | **0.000 ↑** | **0.000 ↑** | **0.000 ↑** | **0.000 ↓** | 0.017 | 0.365 | 0.338 | **0.003 ↑** | **0.000 ↑** | **0.000 ↑** | **0.000 ↓** |
| 6 wk pp to 26 wk pp (cases) | n.d. | n.d. | n.d. | n.d. | n.d. | n.d. | 0.203 | n.d. | n.d. | 0.025 | **0.000 ↓** | n.d. | n.d. |
| 1st trim to 3rd trim (controls) | 0.750 | 0.007 | 0.855 | 0.116 | 0.012 | 0.280 | **0.003 ↑** | 0.274 | 0.098 | **0.004 ↑** | **0.000 ↑** | **0.001 ↓** | **0.003 ↑** |
| 3rd trim to 6 wk pp (controls) | 0.285 | 0.957 | 0.567 | 0.430 | 0.770 | **0.001 ↓** | 0.024 | 0.854 | 0.927 | **0.001 ↑** | **0.000 ↑** | **0.001 ↑** | **0.003 ↓** |
| 3rd trim to 26 wk pp (controls) | 0.885 | 0.943 | 0.291 | 0.195 | 0.981 | **0.002 ↓** | 0.341 | 0.675 | 0.361 | 0.381 | 0.058 | **0.001 ↑** | **0.001 ↓** |
| 6 wk pp to 26 wk pp (controls) | n.d. | n.d. | n.d. | n.d. | n.d. | n.d. | 0.009 | n.d. | n.d. | 0.005 | **0.000 ↓** | n.d. | n.d. |

^*^ Abbreviations used: pre-conc. = pre-conception; trim = trimester; wk pp = weeks postpartum; GalNAc = *N*-acetylgalactosamine; Gal = galactose; SA = sialic acid; Bis = Bisection; Fuc = fucosylation; Tri = triantennary glycans.

| **Supplementary Table S3** Means and standard errors of the IgA *N*- and *O*-glycosylation traits at each time point for both RA patients (Cases) and healthy controls. | | | | | | | | | | | | | | |
| --- | --- | --- | --- | --- | --- | --- | --- | --- | --- | --- | --- | --- | --- | --- |
|  |  |  | Controls | | Cases | |  |  |  |  | Controls | | Cases | |
|  |  |  | Mean | SEM | Mean | SEM |  |  | |  | Mean | SEM | Mean | SEM |
| ***O*-glycosylation** | # GalNAc | pre-conc.^*^ | n.d. | n.d. | 4.81 | 0.00 | *N*-glycosylation site **Asn340** | intact | % SA | pre-conc. | n.d. | n.d. | 95.12 | 0.14 |
|  |  | 1st trim | 4.81 | 0.01 | 4.80 | 0.00 |  |  |  | 1st trim | 95.43 | 0.30 | 94.83 | 0.11 |
|  |  | 2nd trim | 4.81 | 0.01 | 4.81 | 0.00 |  |  |  | 2nd trim | 95.37 | 0.30 | 95.14 | 0.11 |
|  |  | 3rd trim | 4.81 | 0.01 | 4.82 | 0.00 |  |  |  | 3rd trim | 95.62 | 0.30 | 95.25 | 0.11 |
|  |  | 6 wk pp | 4.84 | 0.01 | 4.82 | 0.00 |  |  |  | 6 wk pp | 95.51 | 0.30 | 95.15 | 0.11 |
|  |  | 12 wk pp | 4.82 | 0.01 | 4.82 | 0.00 |  |  |  | 12 wk pp | 95.70 | 0.30 | 95.12 | 0.11 |
|  |  | 26 wk pp | 4.81 | 0.01 | 4.82 | 0.00 |  |  |  | 26 wk pp | 95.65 | 0.31 | 95.14 | 0.11 |
|  | # Gal | pre-conc. | n.d. | n.d. | 4.02 | 0.01 |  | trunc. | % SA | pre-conc. | n.d. | n.d. | 89.96 | 0.11 |
|  |  | 1st trim | 3.96 | 0.02 | 4.00 | 0.01 |  |  |  | 1st trim | 89.94 | 0.23 | 90.26 | 0.09 |
|  |  | 2nd trim | 3.96 | 0.02 | 4.00 | 0.01 |  |  |  | 2nd trim | 89.67 | 0.23 | 90.34 | 0.09 |
|  |  | 3rd trim | 3.99 | 0.02 | 4.01 | 0.01 |  |  |  | 3rd trim | 89.65 | 0.23 | 90.24 | 0.09 |
|  |  | 6 wk pp | 3.99 | 0.02 | 4.04 | 0.01 |  |  |  | 6 wk pp | 89.67 | 0.23 | 89.87 | 0.09 |
|  |  | 12 wk pp | 3.98 | 0.02 | 4.03 | 0.01 |  |  |  | 12 wk pp | 89.58 | 0.23 | 89.96 | 0.09 |
|  |  | 26 wk pp | 3.99 | 0.02 | 4.03 | 0.01 |  |  |  | 26 wk pp | 89.79 | 0.24 | 90.14 | 0.09 |
|  | # SA | pre-conc. | n.d. | n.d. | 3.10 | 0.02 |  | intact | % Bis | pre-conc. | n.d. | n.d. | 59.42 | 0.58 |
|  |  | 1st trim | 3.04 | 0.04 | 3.11 | 0.01 |  |  |  | 1st trim | 53.37 | 1.36 | 56.73 | 0.49 |
|  |  | 2nd trim | 3.04 | 0.04 | 3.13 | 0.01 |  |  |  | 2nd trim | 53.89 | 1.35 | 57.59 | 0.49 |
|  |  | 3rd trim | 3.02 | 0.04 | 3.09 | 0.01 |  |  |  | 3rd trim | 55.78 | 1.35 | 59.26 | 0.48 |
|  |  | 6 wk pp | 3.03 | 0.04 | 3.12 | 0.01 |  |  |  | 6 wk pp | 59.15 | 1.35 | 61.50 | 0.49 |
|  |  | 12 wk pp | 3.07 | 0.04 | 3.11 | 0.01 |  |  |  | 12 wk pp | 57.66 | 1.35 | 60.60 | 0.48 |
|  |  | 26 wk pp | 3.04 | 0.04 | 3.15 | 0.01 |  |  |  | 26 wk pp | 57.54 | 1.39 | 60.55 | 0.48 |
|  | SA per Gal | pre-conc. | n.d. | n.d. | 0.77 | 0.00 |  | trunc. | % Bis | pre-conc. | n.d. | n.d. | 55.91 | 0.44 |
|  |  | 1st trim | 0.77 | 0.01 | 0.78 | 0.00 |  |  |  | 1st trim | 51.96 | 1.10 | 53.74 | 0.40 |
|  |  | 2nd trim | 0.77 | 0.01 | 0.78 | 0.00 |  |  |  | 2nd trim | 53.07 | 1.10 | 53.55 | 0.39 |
|  |  | 3rd trim | 0.76 | 0.01 | 0.77 | 0.00 |  |  |  | 3rd trim | 54.39 | 1.10 | 55.16 | 0.39 |
|  |  | 6 wk pp | 0.76 | 0.01 | 0.77 | 0.00 |  |  |  | 6 wk pp | 58.76 | 1.11 | 58.95 | 0.39 |
|  |  | 12 wk pp | 0.77 | 0.01 | 0.77 | 0.00 |  |  |  | 12 wk pp | 57.25 | 1.10 | 57.92 | 0.39 |
|  |  | 26 wk pp | 0.76 | 0.01 | 0.78 | 0.00 |  |  |  | 26 wk pp | 55.81 | 1.11 | 57.49 | 0.39 |
|  | Gal per GalNAc | pre-conc. | n.d. | n.d. | 0.84 | 0.00 |  | trunc. | % Fuc | pre-conc. | n.d. | n.d. | 92.95 | 0.18 |
|  |  | 1st trim | 0.82 | 0.00 | 0.83 | 0.00 |  |  |  | 1st trim | 93.21 | 0.36 | 92.69 | 0.14 |
|  |  | 2nd trim | 0.82 | 0.00 | 0.83 | 0.00 |  |  |  | 2nd trim | 92.39 | 0.36 | 92.52 | 0.13 |
|  |  | 3rd trim | 0.83 | 0.00 | 0.83 | 0.00 |  |  |  | 3rd trim | 92.50 | 0.36 | 92.39 | 0.13 |
|  |  | 6 wk pp | 0.82 | 0.00 | 0.84 | 0.00 |  |  |  | 6 wk pp | 93.24 | 0.36 | 92.82 | 0.13 |
|  |  | 12 wk pp | 0.83 | 0.00 | 0.84 | 0.00 |  |  |  | 12 wk pp | 93.06 | 0.36 | 92.99 | 0.13 |
|  |  | 26 wk pp | 0.83 | 0.00 | 0.84 | 0.00 |  |  |  | 26 wk pp | 93.48 | 0.37 | 92.93 | 0.13 |
| *N*-glycosylation site **Asn144** | % SA | pre-conc. | n.d. | n.d. | 58.37 | 0.49 |  | trunc. | % Tri | pre-conc. | n.d. | n.d. | 5.10 | 0.15 |
|  |  | 1st trim | 62.58 | 1.00 | 59.72 | 0.39 |  |  |  | 1st trim | 5.70 | 0.31 | 5.29 | 0.12 |
|  |  | 2nd trim | 64.61 | 1.08 | 60.55 | 0.38 |  |  |  | 2nd trim | 6.63 | 0.31 | 5.53 | 0.11 |
|  |  | 3rd trim | 63.78 | 1.01 | 60.46 | 0.39 |  |  |  | 3rd trim | 6.27 | 0.31 | 5.49 | 0.11 |
|  |  | 6 wk pp | 60.46 | 1.03 | 57.75 | 0.38 |  |  |  | 6 wk pp | 5.65 | 0.31 | 5.19 | 0.11 |
|  |  | 12 wk pp | 59.94 | 1.06 | 58.03 | 0.38 |  |  |  | 12 wk pp | 5.77 | 0.31 | 4.92 | 0.11 |
|  |  | 26 wk pp | 60.42 | 1.01 | 57.89 | 0.38 |  |  |  | 26 wk pp | 5.40 | 0.31 | 4.96 | 0.11 |
|  | % Bis | pre-conc. | n.d. | n.d. | 29.56 | 0.56 |  |  |  |  |  |  |  |  |
|  |  | 1st trim | 24.85 | 1.33 | 28.46 | 0.49 |  |  |  |  |  |  |  |  |
|  |  | 2nd trim | 25.79 | 1.38 | 29.68 | 0.48 |  |  |  |  |  |  |  |  |
|  |  | 3rd trim | 26.91 | 1.34 | 31.10 | 0.49 |  |  |  |  |  |  |  |  |
|  |  | 6 wk pp | 28.57 | 1.35 | 32.49 | 0.48 |  |  |  |  |  |  |  |  |
|  |  | 12 wk pp | 27.24 | 1.37 | 32.11 | 0.48 |  |  |  |  |  |  |  |  |
|  |  | 26 wk pp | 26.17 | 1.34 | 32.02 | 0.48 |  |  |  |  |  |  |  |  |

^*^ Abbreviations used: pre-conc. = pre-conception; trim = trimester; wk pp = weeks postpartum; GalNAc = N-acetylgalactosamine; Gal = galactose; SA = sialic acid; Bis = Bisection; Fuc = fucosylation; Tri = triantennary glycans; SEM = standard error of the mean.
