# Supplementary figures and images for: IgA N- and O-glycosylation profiling reveals no association with the pregnancy-related improvement in rheumatoid arthritis
Source: Arthritis Res Ther. 2017 Jul 5;19:160. doi: 10.1186/s13075-017-1367-0 (PMC5498977; doi:10.1186/s13075-017-1367-0)

Additional File 4


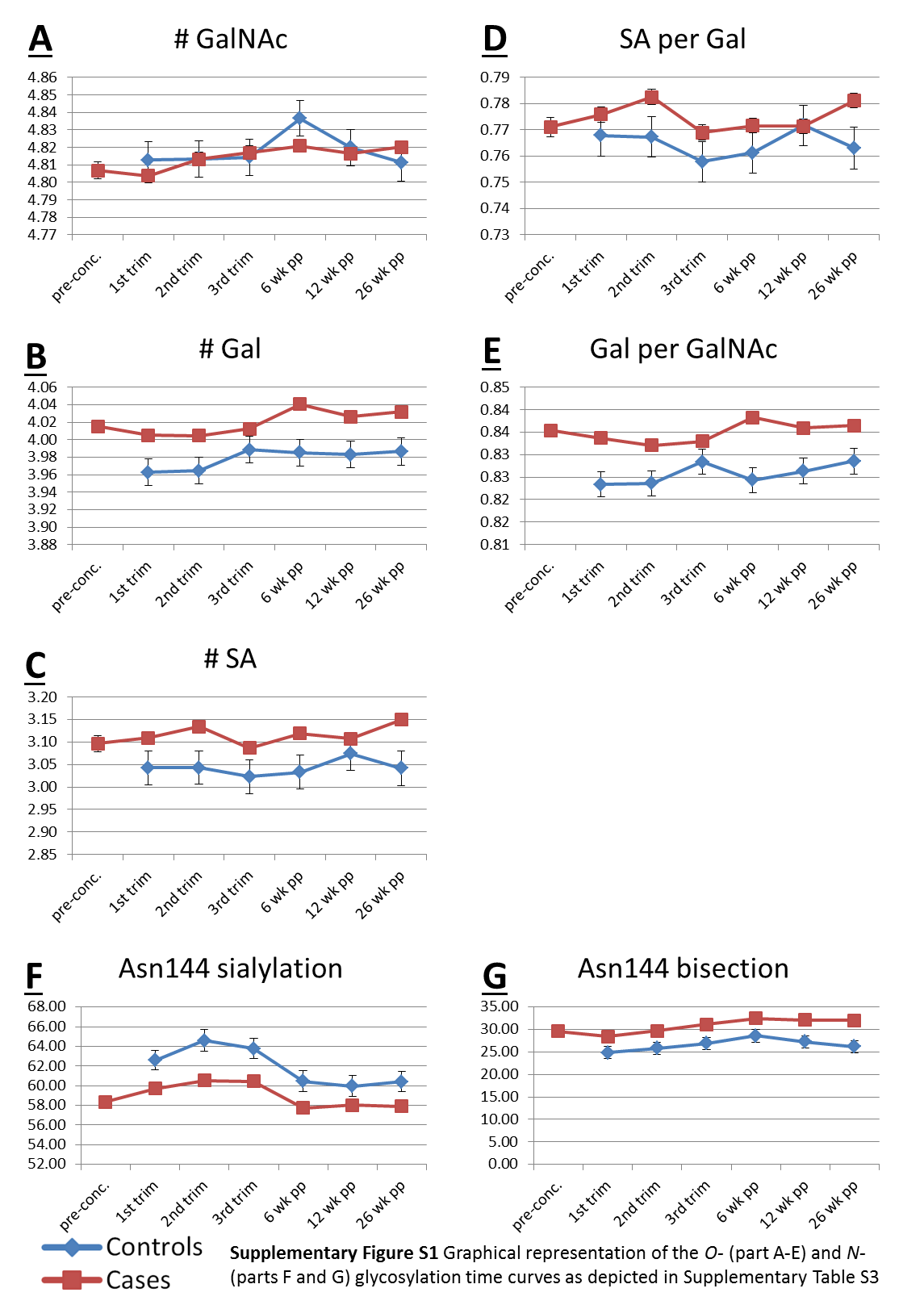

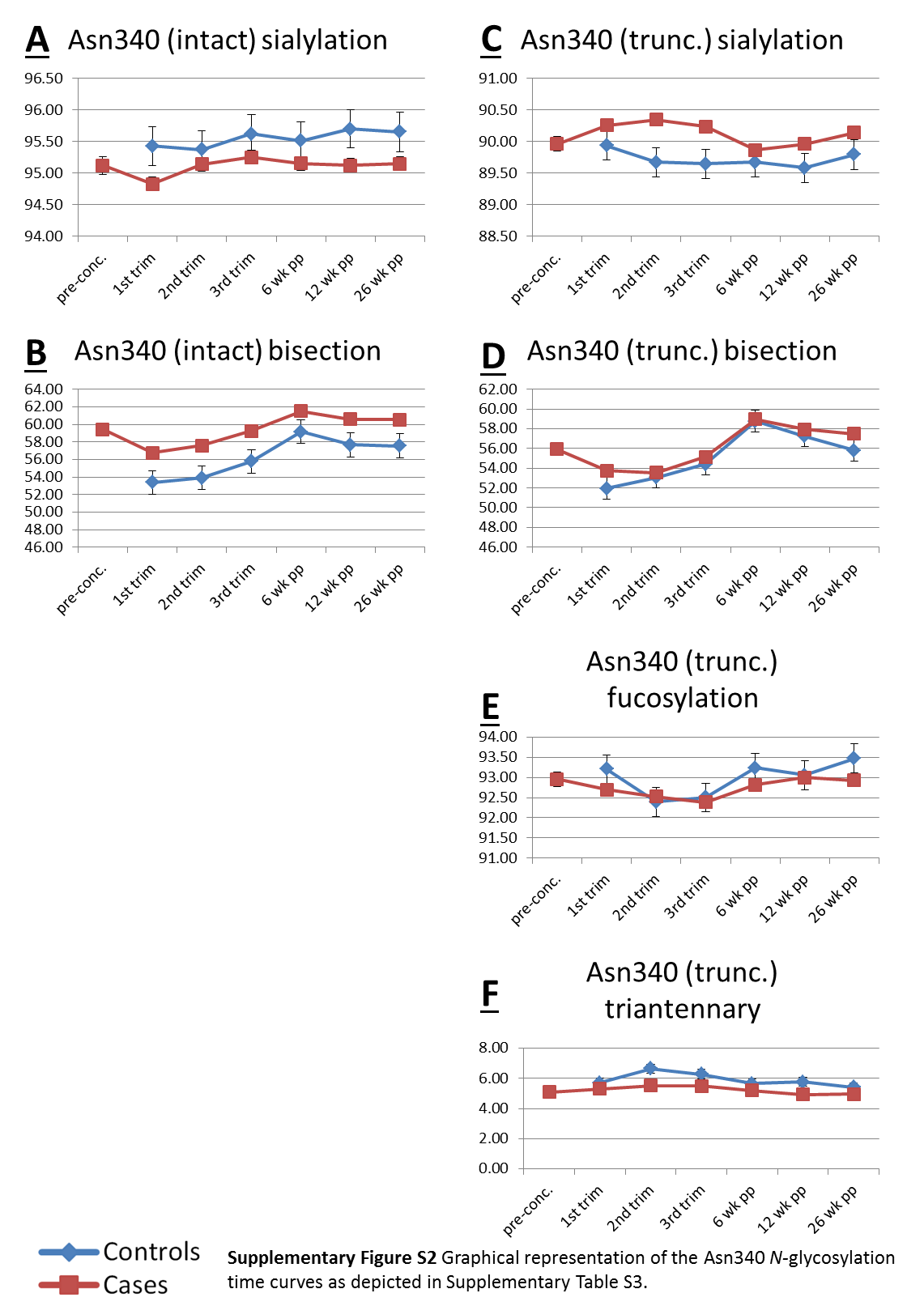

Supplement: Supplementary file 3 — Supplementary figures. Figures S1 and S2 Values depicted in graphs from data in Additional file 2: Table S3. (DOCX 248 kb) [file 13075_2017_1367_MOESM3_ESM.docx]
