# Supplementary material for: IgA N- and O-glycosylation profiling reveals no association with the pregnancy-related improvement in rheumatoid arthritis
Source: Arthritis Res Ther. 2017 Jul 5;19:160. doi: 10.1186/s13075-017-1367-0 (PMC5498977; doi:10.1186/s13075-017-1367-0)
Supplement: Supplementary file 4 — Supplementary results. Results for statistical comparison of IgG and IgA glycosylation association with disease activity. (DOCX 13 kb) [file 13075_2017_1367_MOESM4_ESM.docx]

Additional File 2

# Supplementary Results

## DAS28 is predominantly associated with IgG glycosylation

The association between disease activity and glycosylation was explored using a bootstrap analysis of the backward elimination procedure for multivariate regression analysis. The analysis revealed that especially IgG sialylation was often included in the models for predicting RA disease activity. Interestingly, while the IgG1 sialylation traits are primarily (60-66% of the total counts) included after delivery, the combined IgG2 and IgG3 sialylation traits are primarily (69-76%) included in pregnancy-associated models. Furthermore, IgA O-glycan numbers of GalNAcs and galactoses are regularly included, with distributions comparable to IgG1 sialylation traits. Sialylation of IgA Asn144 and bisection of IgA Asn340 were also found among the top hits, but no difference in scoring during or after delivery was observed.

Indeed, when the variables obtained from the bootstrap procedure are incorporated in multivariate models using the disease activity as the dependent variable highly significant models are generated. At the first trimester IgG2/3 sialylation (β=-0.6; p=1.9e-3) and sialic acid per galactose (β=0.5; p=2.5e-4) are associated with the disease activity (model p=0.0000; R^2^=0.32). At the second trimester (model p=0.0000; R^2^=0.37) IgG1 sialylation (β=2.4; p=0.029), IgG1 galactosylation (β=-1.7; p=0.011), and IgG2/3 sialylation (β=-0.5; p=0.036) are associated with disease activity, as well as Asn340 bisection (β=0.2; p=0.043). Remarkably, at the third trimester (model p=0.0000; R^2^=0.36) the use of prednisone (β=0.3; p=8.9e-5), autoantibody positivity (β=0.2; p=0.019), and the presence of triantennary glycans at IgA Asn340 (β=0.2; p=0.043) show positive associations with disease activity, and a negative association was observed for bisection at Asn340 (β=-0.3; p=0.013).

Six weeks after delivery none of the included covariates is significantly associating with disease activity, although the model has a p-value of 0.0003 and an R^2^ of 0.26. However, twelve weeks after delivery (model p=0.0000; R^2^=0.26) IgG1 sialylation (β=-0.6; p=7.3e-4) and sialic acid per galactose (β=0.5; p=1.2e-4) were found to associate with disease activity, similar to the first trimester of pregnancy, although on a different subclass. At six months after delivery IgG1 sialylation (β=-0.8; p=5.9e-3) was negatively associated with disease activity in a model with an R^2^ of 0.27 (p=0.0005).

1. Bondt A, Nicolardi S, Jansen BC, et al. Longitudinal monitoring of immunoglobulin A glycosylation during pregnancy by simultaneous MALDI-FTICR-MS analysis of N- and O-glycopeptides. *Sci Rep* 2016;6:27955. doi: 10.1038/srep27955
